# P-677. Title: Estimation of Respiratory Syncytial Virus-attributable Hospitalizations among Older Adults in Japan between 2015 and 2018: an Administrative Health Claims Database Analysis

**DOI:** 10.1093/ofid/ofae631.873

**Published:** 2025-01-29

**Authors:** Masafumi Seki, Yasuhiro Kobayashi, Estelle Meroc, Takahiro Kitano, Aleksandra Polkowska-Kramek, Asuka Yoshida, Caihua Liang, Robin Bruyndonckx, Solomon Molalign Moges, Eduardo Conde-Sousa, Charles Nuttens, Bradford D Gessner, Elizabeth Begier

**Affiliations:** Saitama Medical University International Medical Center, Hidaka City, Saitama, Japan; Pfizer Japan, Shibuya-ku, Tokyo, Japan; P95 Pharmacovigilance and Epidemiology Services, Leuven, Belgium, Leuven, Vlaams-Brabant, Belgium; Pfizer Japan Inc., Tokyo, Tokyo, Japan; P95, Leuven, Brabant Wallon, Belgium; Pfizer Japan Inc., Tokyo, Tokyo, Japan; Pfizer Inc, New York, New York; P95, Leuven, Brabant Wallon, Belgium; P-95, KESSEL-LO (LEUVEN), Vlaams-Brabant, Belgium; P95 Pharmacovigilance and Epidemiology, Leuven, Belgium, Porto, Porto, Portugal; Pfizer Inc, New York, New York; Pfizer Biopharma Group, Collegeville, Pennsylvania; Pfizer Vaccines, Dublin, Dublin, Ireland

## Abstract

**Background:**

Respiratory Syncytial Virus (RSV) incidence in adults is often underestimated, mainly because of infrequent testing, nonspecific symptoms and lower diagnostic test sensitivity compared to infants. We estimated population-based incidence rates (IRs) of RSV-attributable hospitalizations in adults ≥60 years in Japan using a retrospective time-series model-based approach.

**Figure:**
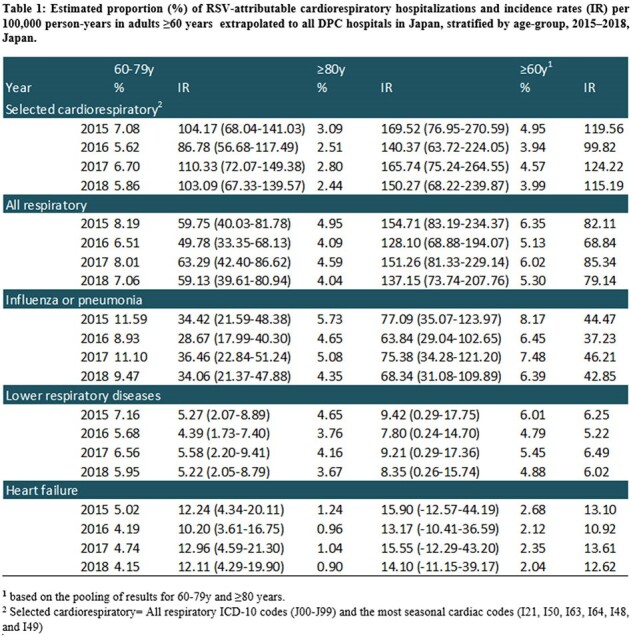

**Methods:**

We obtained hospitalization data from the Medical Data Vision (MDV) database, which is restricted to Diagnosis Procedure Combination (DPC) hospitals; data were included for 01/01/2015-31/12/2018, with 2018 the last complete year available before COVID-19. We estimated the yearly and age-specific RSV-attributable IR of hospitalizations for five cardiorespiratory outcomes defined based on selected ICD-10 codes. We used a quasi-Poisson regression model accounting for periodic and aperiodic time trends, as well as viral activity proxies. An offset was included in the model to account for the increase of the source database size. We extrapolated our results to all DPC hospitals in Japan.

**Results:**

In adults aged ≥60 years, the IRs of RSV-attributable cardiorespiratory hospitalizations were 100-124 per 100,000 person-years (up to 7% of selected cardiorespiratory hospitalizations) and 69-85 per 100,000 person-years for respiratory-specific hospitalizations (up to 8% of all respiratory hospitalizations). IRs for all outcomes were higher in adults aged ≥80 years compared to those aged 60-79 years (Table 1).

**Conclusion:**

Modeled RSV-attributable IRs in Japan were lower than in most other developed countries, likely due to the MDV database only including advanced treatment hospitals. Taking this into account, our results indicate a high burden of RSV-attributable hospitalizations in older adults in Japan, highlighting the need to implement effective RSV prevention strategies. Future work will include a sensitivity analysis extrapolating IRs to all hospitals in Japan.

**Disclosures:**

**Yasuhiro Kobayashi, MS**, Pfizer Japan Inc: employee of Pfizer Japan Inc.|Pfizer Japan Inc: Stocks/Bonds (Private Company) **Aleksandra Polkowska-Kramek, n/a**, Pfizer: employee of P95 which received funding from Pfizer to conduct this study **Caihua Liang, MD, PhD**, Pfizer: Stocks/Bonds (Private Company) **Charles Nuttens, n/a**, Pfizer: Stocks/Bonds (Private Company) **Bradford D. Gessner, M.D., M.P.H.**, Pfizer: Employee|Pfizer: Stocks/Bonds (Public Company) **Elizabeth Begier, MD, M.P.H.**, Pfizer Vaccines: Employee|Pfizer Vaccines: Stocks/Bonds (Private Company)

